# Dysbiosis: The first hit for digestive system cancer

**DOI:** 10.3389/fphys.2022.1040991

**Published:** 2022-11-22

**Authors:** Si Mei, Zhe Deng, Yating Chen, Dimin Ning, Yinmei Guo, Xingxing Fan, Ruoyu Wang, Yuelin Meng, Qing Zhou, Xuefei Tian

**Affiliations:** ^1^ Department of Physiology, Faculty of Medicine, Hunan University of Chinese Medicine, Changsha, Hunan, China; ^2^ Hunan Key Laboratory of Translational Research in Formulas and Zheng of Traditional Chinese Medicine, Hunan University of Chinese Medicine, Changsha, Hunan, China; ^3^ Department of Internal Medicine, College of Integrated Chinese and Western Medicine, Hunan University of Chinese Medicine, Changsha, Hunan, China; ^4^ State Key Laboratory of Quality Research in Chinese Medicine, Macau Institute for Applied Research in Medicine and Health, Macau University of Science and Technology, Macau, Macau SAR, China; ^5^ Department of Liver Diseases, The First Hospital of Hunan University of Chinese Medicine, Changsha, Hunan, China; ^6^ Department of Andrology, The First Hospital of Hunan University of Chinese Medicine, Changsha, Hunan, China

**Keywords:** gut microbiota, dysbiosis, colorectal cancer, hepatocellular carcinoma, carcinogenesis

## Abstract

Gastrointestinal cancer may be associated with dysbiosis, which is characterized by an alteration of the gut microbiota. Understanding the role of gut microbiota in the development of gastrointestinal cancer is useful for cancer prevention and gut microbiota-based therapy. However, the potential role of dysbiosis in the onset of tumorigenesis is not fully understood. While accumulating evidence has demonstrated the presence of dysbiosis in the intestinal microbiota of both healthy individuals and patients with various digestive system diseases, severe dysbiosis is often present in patients with digestive system cancer. Importantly, specific bacteria have been isolated from the fecal samples of these patients. Thus, the association between dysbiosis and the development of digestive system cancer cannot be ignored. A new model describing this relationship must be established. In this review, we postulate that dysbiosis serves as the first hit for the development of digestive system cancer. Dysbiosis-induced alterations, including inflammation, aberrant immune response, bacteria-produced genotoxins, and cellular stress response associated with genetic, epigenetic, and/or neoplastic changes, are second hits that speed carcinogenesis. This review explains the mechanisms for these four pathways and discusses gut microbiota-based therapies. The content included in this review will shed light on gut microbiota-based strategies for cancer prevention and therapy.

## Introduction

Dysbiosis, defined as perturbations in the quality or quantity of gut microbiota, has recently emerged as a crucial pathophysiological factor for cancer onset and progression, especially digestive system cancer (Ponziani et al., 2019; Sobhani et al., 2019; Yachida et al., 2019; Huang et al., 2020; Jia et al., 2021; Yang et al., 2021; Kartal et al., 2022). Unlike gastric cancer and cervical cancer, which are induced by single pathogenic agents, the pattern of gut microbiota-associated carcinogenesis remains elusive. *Helicobacter pylori* (Hatakeyama, 2014; Amieva and Peek, 2016) and human papillomavirus (HPV) (Marx, 1986; Clifford et al., 2017) are widely accepted as the etiologic agents of gastric cancer and cervical cancer, respectively. Long-term infection by *H. pylori* is the strongest risk factor for both intestinal and diffuse gastric non-cardia carcinomas. In addition, *H. pylori* eradication is the standard therapy for gastric cancer prevention (Yan et al., 2022). Similarly, persistent infection with oncogenic HPV types is strongly associated with the development of cervical cancer. HPV vaccination is an effective method of cervical cancer prevention (Wang et al., 2020a; Printz, 2021) to significantly reduce morbidity and mortality due to cervical cancer. Therefore, the causality of microorganisms demonstrated in gastric cancer and cervical cancer leads us to consider the possible roles of gut microbiota in digestive system cancer.

In the clinic, only a few bacterial species have been associated with increased cancer morbidity, including *Fusobacterium nucleatum* (Yu et al., 2017; Kong et al., 2021; Chen et al., 2022). Monitoring GI microbiota is also not included in therapeutic guidelines for digestive system cancer, although it is recommended as the standard therapy for *Clostridioides difficile* infection in adults (Johnson et al., 2021; van Prehn et al., 2021). Despite this, a strong correlation exists between dysbiosis and digestive system cancer carcinogenesis, as confirmed by numerous preclinical and clinical studies. Composition shifts of gut microbiota have been reported in patients with colorectal cancer (CRC) (Castellarin et al., 2012; Yachida et al., 2019; Jia et al., 2021; Yang et al., 2021) and hepatocellular carcinoma (HCC) (Jia et al., 2021; Ray, 2021; Schneider et al., 2022). Fecal transplantation from CRC model mice to control mice identified cancer-related biochemical or behavioral changes in the recipient (Wong et al., 2017; Li et al., 2019; Chang et al., 2020). These findings lead to questions regarding the potential role of dysbiosis on the onset of digestive system cancer. Based on collective evidence, we propose that dysbiosis works as the first hit on the development of digestive system cancer. The presence of dysbiosis (the first hit) will not guarantee carcinogenesis unless dysbiosis-associated alterations (second hits) also occur. This review discusses the presence of dysbiosis in digestive system cancer and how dysbiosis-associated alterations can potentiate carcinogenesis. Microbiota-based cancer therapies are also described.

## Normal gut microbiota

Trillions of microbes have been identified in the normal adult digestive system tract, including facultative anaerobes (e.g., *Lactobacilli* and *Enterobacteria*) and strict anaerobes (e.g., *Bacteroides* and *Bifidobacterium*) (Lloyd-Price et al., 2016). Firmicutes and Bacteroidetes are major phyla in a healthy gut (>90%). Other phyla include Proteobacteria (<5%), Actinobacteria (<2%), Verrucomicrobia*,* and Fusobacteria (<1%), and others. The gut microbiota is dynamic and varies among individuals. Furthermore, gut microbiota can be influenced by environmental factors, diet, and medications (Zmora et al., 2019; Lindell et al., 2022). Recent research has demonstrated the important role gut microbiota play in several physiological processes, including immune system development and maturation (Rooks and Garrett, 2016; Song et al., 2020; Paik et al., 2022), bile acid metabolism (Funabashi et al., 2020; Poland and Flynn, 2021), energy consumption (Coelho et al., 2019; Miyamoto et al., 2019), and neurotransmitter biosynthesis (Yano et al., 2015; Choi et al., 2020). Therefore, dysbiosis may contribute to various diseases, including digestive system cancer. As aforementioned, while no single genus has been associated with intestinal cancer, dysbiosis of gut microbiota has been observed and may be the first hit for gastrointestinal cancer. Thus, this dysbiosis may be a better diagnostic marker or a potential therapeutic target.

## The first hit: Perturbance of gut microbiota in digestive system cancer

### Dysbiosis of gut microbiota in CRC

CRC is a heterogeneous group of cancers that mostly develop from polyps (neoplastic precursor lesions). CRC accounted for approximately 10% of all diagnosed cancers and cancer-related deaths worldwide in 2020 (Sung et al., 2021). Hereditary (e.g., hereditary colorectal cancer syndromes and *APC* mutations) and environmental risk factors (e.g., low intake of vegetables and fruits, high body fat, and obesity) play roles in CRC development. Male sex and increasing age are also positively associated with CRC incidence.

Recently, microbiome profiling *via* 16S rRNA or shotgun metagenomics of stool samples confirmed dysbiosis in patients with CRC (Dai et al., 2018; Thomas et al., 2019; Coker et al., 2022). The representation of three bacterial species is consistently increased in patients with CRC, including *F. nucleatum*, *Enterotoxigenic bacteroides fragilis* (*ETBF*), and *Escherichia coli* with the *pks* genetic island (*pks* + *E. coli*) (Table 1). Other non-specific genera include at least 27 genera, including *Porphyromonas*, *Peptostreptococcus*, and *Prevotella,* and the order Clostridiales (Wirbel et al., 2019). In addition, dysbiosis also relates to certain precursor lesions in CRC, including the adenocarcinoma (70–90%) and serrated neoplasia (10–20%) pathways. Clinical tests have shown significant decreases in microbial diversity, overall composition, and normalized taxon abundance in these two precursors, especially in patients with advanced traditional adenoma-carcinoma (Peters et al., 2016). *Clostridia* operational taxonomic units are depleted in these patients, whereas classes Gammaproteobacteria and Bacilli, order Enterobacteriales, and genera *Streptococcus* and *Actinomyces* were enriched. Therefore, some intestinal bacteria may be biomarkers for CRC. Multi-omics analysis from a CRC cohort showed a significantly increased abundance of *F. nucleatum* spp. from intramucosal carcinoma to more advanced stages, whereas *Atopobium parvulum* and *Actinomyces odontolyticus* were only dominant in multiple polypoid adenomas and/or intramucosal carcinomas (Yachida et al., 2019).

**TABLE 1:** CRC-associated bacteria and their effects on carcinogenesis.

Bacteria	Effects on carcinogenesis	References
*Fusobacterium nucleatum* (*F. nucleatum*)	Promotes CRC cell proliferation *in vitro*	Castellarin et al. (2012); Kostic et al. (2012)
	Increases tumor growth rates in patient-derived CRC xenografts in mice	Bullman et al. (2017)
	Increase levels of lymphocyte-attracting chemokines CCL5, CCL20, and CXCL11	Cremonesi et al. (2018)
	Recruit other bacteria to form biofilms coating human CRCs	Dejea et al. (2018)
*Enterotoxigenic Bacteroides fragilis (ETBF)*	Promote the development of precancerous lesions (i.e., adenomas)	Dejea et al. (2018)
	Induce a pro-carcinogenic Th17 response by recruiting M-MDSCs	Wu et al. (2009)
	Induce DNA damage *via* promoting the inflammation and oxidative stress	Irrazabal et al. (2020)
	Induced the expression and secretion of CXCL1-ortholog IL-8 from epithelial cells *via* activation of NF-κB	Kim et al. (2001)
	Enhance tumorigenesis in preclinical CRC models	Arthur et al. (2012)
*Escherichia coli* (*E. coli*)	Produce the genotoxin colibactin and result in mutagenic DNA damage in colonic epithelial cells	Cuevas-Ramos et al. (2010; Irrazabal et al. (2020)
	Induce intestinal stem cell mutations *in vitro*	Pleguezuelos-Manzano et al. (2020)
	Increase levels of lymphocyte-attracting chemokines CCL5, CCL20, and CXCL11	Cremonesi et al. (2018)
	Induce DNA damage *via* promoting inflammation and oxidative stress	Wang et al. (2015)

M-MDSCs, monocytic-like myeloid-derived suppressor cells.

Gut microbiota is also correlated with CRC therapy. In neoadjuvant chemoradiotherapy (nCRT) for locally advanced rectal cancer (LARC), the gut microbiota in patients with effective response differed significantly from those in non-responders (Yi et al., 2021). Some butyrate-producing bacteria, including *Roseburia*, *Dorea*, and *Anaerostipes*, were dominant in responders, whereas non-responders showed increased Coriobacteriaceae and *Fusobacterium*. Gut microbiota can metabolize some of the chemotherapeutic drugs, thus regulating the response to chemotherapy (Chattopadhyay et al., 2021). Bacterial species, such as *F. nucleatum*, are also associated with CRC recurrence (Yi et al., 2021). *F. nucleatum*-positive patients after nCRT treatment showed a depletion of CD8^+^ T cells and may be at a higher risk of recurrence (Serna et al., 2020).

In contrast, some bacterial species may play anti-tumorigenic roles in CRC. *Faecalibaculum rodentium* (*Holdemanella biformis* in humans) reduced tumor growth in a mouse intestinal tumor model by producing short-chain fatty acids (SCFAs) (Zagato et al., 2020). SCFAs control protein acetylation and tumor cell proliferation by suppressing calcineurin/NFATc3 activity. Similarly, oral gavage of *Streptococcus thermophilus* significantly prevented tumor formation in two mouse models of intestinal tumors by activating oxidative phosphorylation and downregulating Hippo pathway kinases (Li et al., 2021). CRC cells co-incubated with *Streptococcus thermophilus* or its conditioned medium showed decreased proliferation rate *via* produced β-galactosidase *in vitro*. The anti-tumor effect of *S. thermophilus* was also attributed to the increased abundance of commensal bacteria such as *Bifidobacterium* and *Lactobacillus*. Other bacterial species showing a protective effect on CRC include *Akkermansia muciniphila* (Fan et al., 2021), Clostridiales (Montalban-Arques et al., 2021), *Lactobacillus reuteri* (Bell et al., 2022), and *Bacillus toyonensis* (Chen et al., 2020).

### Dysbiosis of gut microbiota in HCC

Liver cancer is another global health challenge that includes hepatocellular carcinoma (HCC) and intrahepatic cholangiocarcinoma (iCCA). HCC comprises the majority of primary liver cancer cases (90%) and is the third leading cause of cancer-related deaths worldwide (Sung et al., 2021). It usually develops from a series of risk factors, such as susceptibility genes (*TERT* mutation), viral risk factors (HBV and HCV infection), alcohol-induced liver disease (alcoholic cirrhosis), or non-alcoholic disease (non-alcoholic fatty liver disease, NAFLD).

Accumulating evidence indicates the association of dysbiosis in HCC development (Table 2). A recent long-term, large-scale study identified gut microbiota instability mostly related to factors contributing to metabolic syndrome, such as fatty liver disease (FLD) and diabetes mellitus (Frost et al., 2021). A total increase of facultative pathogens (e.g., Enterobacteriaceae, *Escherichia*, and *Shigella*) was observed in patients with FLD and was more evident in newly developed cases. FLD is a well-known precursor disease for HCC. The gut microbiota also involve in NAFLD development and progression (Borrelli et al., 2018; Lang and Schnabl, 2020; Behary et al., 2021); thus, they are related to NAFLD-induced HCC. One mechanism of NAFLD is closely linked to endogenous alcohol production (autobrewery syndrome or gut fermentation syndrome), which leads to nonalcoholic steatohepatitis (NASH). Intestinal bacteria isolated from patients with NASH showed an increase in *Klebsiella pneumoniae* strains (Yuan et al., 2019) with varied alcohol-producing activities. The close relationship between dysbiosis and HCC precursor diseases (e.g., FLD, cirrhosis, alcohol dependence syndrome, and alcoholic liver cirrhosis) indicates that the tumor-inducible role of dysbiosis is precursor-dependent. In addition, the composition of gut microbiota varies in patients with different types of precursors. In patients with HCC-cirrhosis, the predominant bacteria are *Clostridium* and *CF231* (a member of the Paraprevotellaceae family) (Lapidot et al., 2020), compared to Clostridiales and Bacteroidales in alcohol dependence syndrome and alcoholic liver cirrhosis (Dubinkina et al., 2017).

**TABLE 2:** Dysbiosis in patients with HCC and HCC-related liver diseases.

Bacteria	Conditions	References
Facultative pathogens ↑ (e.g., Enterobacteriaceae, *Escherichia–Shigella*)	Metabolic liver disease (e.g., FLD)	Frost et al. (2021)
*Clostridium* and *CF231* ↑ Alphaproteobacteria ↓	HCC-cirrhosis vs. cirrhotic without HCC	Lapidot et al. (2020)
Enterobacteriaceae and *Streptococcus* ↑ *Akkermansia* ↓	NAFLD-related cirrhosis with or without HCC	Ponziani et al. (2019)
*Bacteroides* and Ruminococcaceae ↑ *Bifidobacterium* ↓	NAFLD-related cirrhosis and HCC	
Clostridiales and Enterobacteriaceae ↑	ADS without cirrhosis	Dubinkina et al. (2017)
Bacteroidales ↑	ADS with cirrhosis	
Proteobacteria ↑	PD-1 non-responders	Zheng et al. (2019)
*Akkermansia muciniphila* and Ruminococcaceae *spp.*↑	PD-1 responders	
Phylum	Early HCC vs. cirrhosis	Ren et al. (2019)
*Actinobacteria* ↑		
*Verrucomicrobia* ↓		
Genus		
*Gemmiger* and *Parabacteroides* ↑		
*Alistipes*, *Phascolarctobacterium*, and *Ruminococcus* ↓		
*Klebsiella* and *Haemophilus* ↑		
*Faecalibacterium*, *Ruminococcus*, and *Ruminoclostridium* ↑	HBV^+^ HCV^−^ HCC vs. HBV^+^ HCC	Liu et al. (2019)
*Escherichia–Shigella*, *Enterococcus* ↓		
Opportunistic pathogens ↑ (e.g., Gammaproteobacteria, Enterobacteriaceae, and Neisseriaceae)	Primary biliary cirrhosis	Lv et al. (2016)
Potential beneficial bacteria ↓ (e.g., *Acidobacteria*, *Lachnobacterium spp.*, and *Bacteroides eggerthii*)		
*Veillonella*, *Megasphaera*, *Dialister*, *Atopobium*, and *Prevotella* ↑	Cirrhosis	Chen et al. (2016)
Enterobacteriaceae ↑	Decompensated cirrhotic vs. compensated cirrhotic	
Enterobacteriaceae and Enterococcaceae ↑	Prior-HE	Bajaj et al. (2015)
*Veillonella* and *Streptococcus* ↑	Cirrhosis	Qin et al. (2014)
Clostridiales ↓		

FLD, fatty liver diseases; ADS, alcohol dependence syndrome; PD-1, programmed cell death protein 1; HE, hepatic encephalopathy.

Preclinical models have also reported the correlation between dysbiosis and HCC. Generally, HCC with icterus was induced by the consumption of soluble fibers in a series of dysbiotic mice. However, germ-free (GF) mice (without gut microbiota) or antibiotics-treated mice (with decreased gut microbiota) were resistant to such diets and HCC was not stimulated (Singh et al., 2018). Depletion of fermenting bacteria by antibiotics or inhibiting fermentation by plant-derived β-acids prevented HCC progression. In another study, antibiotic treatment decreased liver tumor growth in the primary liver and liver metastasis models *by* recruiting CXCL16, a regulator of natural killer T cell (NKT) accumulation (Ma et al., 2018).

### Dysbiosis of gut microbiota in other digestive system cancers

Limited evidence exists regarding dysbiosis and other digestive system cancers, i.e., esophageal cancer, gastric cancer, and pancreatic cancer (PC). In esophageal cancer, oral or esophageal microbiota, instead of gut microbiota, have been recognized as cancer-related microbial factors. Similarly, the effect of gut microbiota on gastric cancer is negligible. However, gut microbiota may crosstalk with *H. pylori* or gastric microbiota, which are carcinogens for gastric cancer. However, the correlation between gut microbiota and PC is a new research area that emerged in 2017. A recent study reported a fecal microbiota signature in pancreatic cancer (Kartal et al., 2022). Thus, unique microbiota may be used as non-invasive biomarkers for the early detection of pancreatic ductal adenocarcinoma (PDAC). However, further studies are needed to confirm this role.

The information above suggests that the order prevalence of dysbiosis and various types of digestive cancer is CRC > HCC > esophageal cancer > gastric cancer > PC, consistent with PubMed search results. Searches for studies on “gut microbiota and CRC” and “gut microbiota and HCC” indexed in PubMed in the last 5 years revealed 1,458 and 262 articles, respectively. These comprise the first and second most common cancer types among digestive system cancer. Therefore, the following sections mainly focus on CRC and HCC.

## The second hit: Dysbiosis-associated alterations potentiate carcinogenesis in the digestive system

To potentiate carcinogenesis in the digestive system, dysbiosis (the first hit) usually works with other dysbiosis-induced alterations (second hits), including inflammation, immune response, bacteria-produced genotoxins, and cellular stress response associated with genetic, epigenetic and/or neoplastic changes. These alterations together with dysbiosis serve as the sequential hits for the digestive system and speed carcinogenesis in the intestine and liver.

### Dysbiosis drives inflammation

Inflammation is an evolutionarily conserved process involving the activation, recruitment, and action of the innate and adaptive immune systems. Initially, inflammation is an essential host defense against pathogens and the regulation of tissue homeostasis (repair, regeneration, and remodeling). In recent past decades, increased attention has been paid to the contribution of inflammation to cancer development and progression. In the intestine and liver, inflammation can be induced by bacteria-derived metabolites, bile acids, and bacterial components.

#### Bacteria-derived metabolites

The major inflammation-related metabolites produced by gut microbes are trimethylamine N-oxide (TMAO) and SCFAs. TMAO is a converted trimethylamine byproduct from the metabolism of dietary phosphatidylcholine, choline, and carnitine. An elevated serum TMAO level is highly related to cancer, especially CRC. In a nested case–control study (Guertin et al., 2017), men with higher serum choline, the precursor of TMAO, had an approximately three-fold higher risk of developing CRC over the ensuing 14 ± 10 years. In an obesity-associated CRC cohort, the composition of gut microbiota confirmed by 16S rRNA gene sequences differs from that of the CRC cohort without obesity (Sánchez-Alcoholado et al., 2020). A higher abundance of opportunistic pathogens was observed in the obesity cohort with an increased TMAO and proinflammatory cytokine IL-1. The dysbiotic bacteria included *Fusobacterium*, *Clostridium*, *Prevotella*, *Desulfovibrio*, and *Enterococcus*. SCFAs were the major products fermented by intestinal bacteria from indigestible dietary components. Acetate, propionate, and butyrate are three major SCFAs. The Bacteroidetes phylum is mainly responsible for acetate and propionate production, whereas the Firmicutes phylum produces butyrate. Generally, SCFAs have a protective effect on cancer. Decreased SCFA production is associated with increased CRC risks in healthy individuals or patients with CRC-related diseases (Ou et al., 2013). SCFAs help to maintain intestinal integrity by suppressing histone deacetylases (HDACs) in colonic epithelial and immune cells, resulting in decreased pro-inflammatory cytokine release (Chang et al., 2014) and increased apoptosis in CRC cells (Buda et al., 2003). Several preclinical studies have confirmed the anti-tumor role of SCFAs in CRC and HCC (Sheng et al., 2017; Tian et al., 2018). The oral gavage of acetate, butyrate, and propionate in a colitis-associated CRC mouse model significantly decreased tumor size. This protective effect depended on the suppression of pro-inflammatory cytokines, including IL-6, TNF-α, and IL-17. Analysis of the disease activity index further confirmed decreased CRC activity in the group administered SCFAs. Similarly, the administration of mixed SCFAs to HBx (an HBV-encoded oncoprotein) transgenic mice prevented HCC development. The model mice with SCFAs showed fewer tumor nodules and increased expression of disabled homolog 2 (DAB2), a tumor suppressor (McBrearty et al., 2021).

#### Bile acids

Bile acids (BAs), especially secondary BAs, are another gut microbiota-produced product associated with CRC and HCC. Generally, primary BAs are synthesized in the liver from cholesterol. BAs are then secreted into the intestinal lumen to undergo further biotransformation by gut microbiota. In the intestine, BAs are unconjugated by intestinal bacteria-produced bile salt hydrolase (BSH) and biotransformed into secondary BAs *via* bacteria-mediated 7α-dehydroxylation or epimerization. Therefore, the gut microbiota is directly involved in BA biosynthesis and dysbiosis-related alterations in BA composition are related to intestinal and hepatic inflammation. CRC and HCC cohorts both showed close relationships between circulating BAs and carcinogenesis. In one CRC cohort (569 CRC cases and 569 matched controls) (Kühn et al., 2020), a higher CRC risk was associated with increased serum levels of conjugated BA metabolites, including glycocholic acid (GCA), taurochenodeoxycholic acid (TCDCA), taurocholic acid (TCA), glycohyocholic acid (GHCA), glycochenodeoxycholic acid (GCDCA), glycodeoxycholic acid (GDCA), and taurodeoxycholic acid (TDCA). In an HCC cohort (233 pairs of HCC cases and controls), a positive correlation was observed between HCC and overall serum BAs and taurine- or choline-conjugated BAs (Stepien et al., 2021). Increased deoxycholic acid (DCA) has a deleterious effect on intestinal cancer cells by promoting the production of pro-inflammatory cytokines (Liu et al., 2018). Moreover, oral gavage of DCA in a mouse model of intestinal tumors resulted in significant increases in adenoma number and size. Elevated levels of pro-inflammatory cytokines (e.g., IL-1β, IL-6, and TNF-α) and NLRP3 inflammasome-associated proteins (e.g., NOD-like receptor family) were also observed in the DCA-treated group.

**FIGURE 1:**
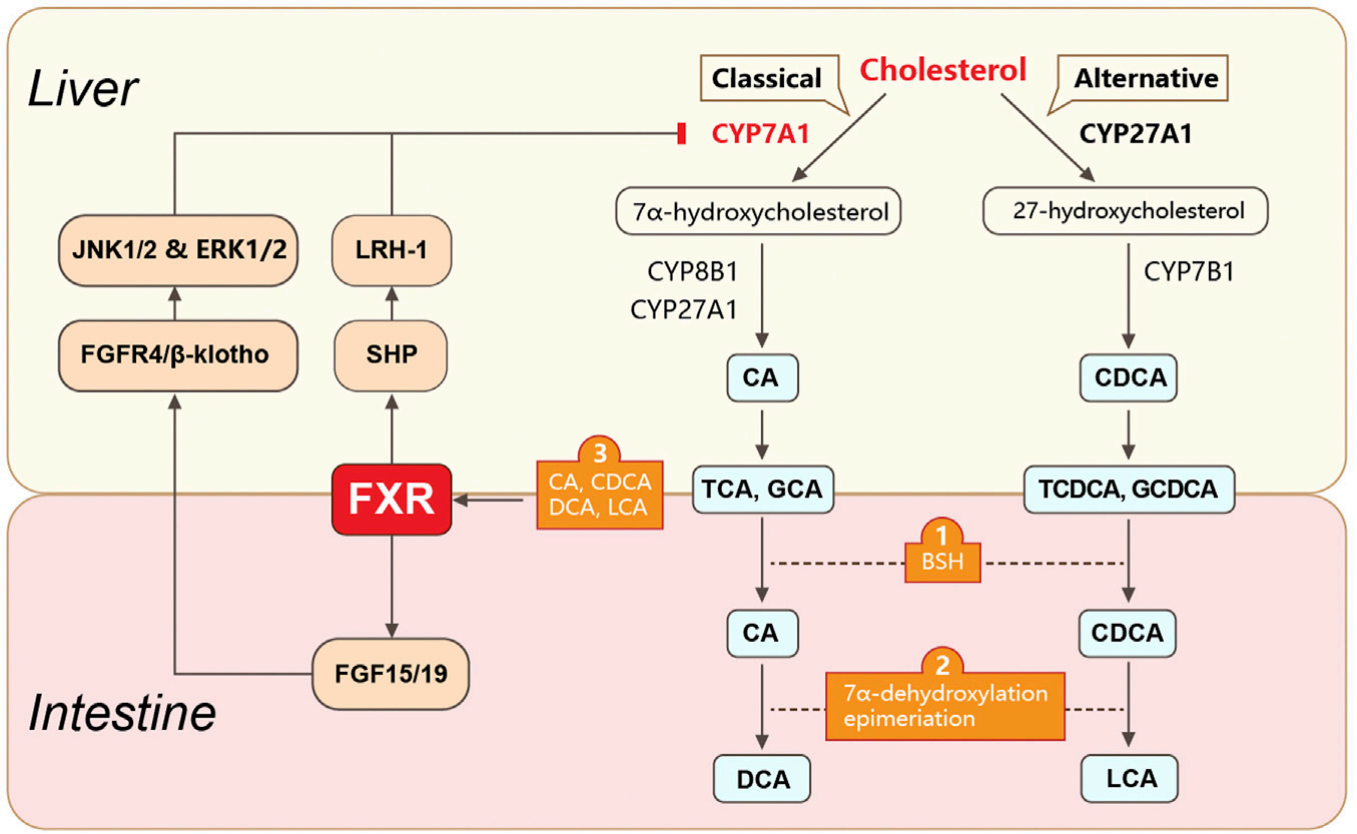
Gut microbiota-involved biosynthesis and metabolism of bile acids. Gut microbiota play roles in BA biosynthesis and metabolism. Primary BAs are synthesized in hepatocytes from cholesterol *via* classical or alternative pathways. They are further conjugated with taurine (in mice) or glycine (in humans) and transformed into conjugated BAs (e.g., TCA, GCA, TCDCA, and GCDCA). The conjugated primary BAs are secreted from the liver into the bile and subsequently into the intestinal lumen, where they are unconjugated by bacteria-produced BSH. The unconjugated primary BAs are biotransformed into secondary BAs (e.g., DCA, UDCA, and LCA) *via* 7α-dehydroxylation or epimerization. The gut microbiota also mediates the enterohepatic circulation of BAs *via* FXR. FXR is activated by unconjugated BAs (e.g., CDCA, DCA, LCA, and CA) and regulates BA synthesis *via* molecules in the liver and intestine. In the liver, FXR suppresses the expression of CYP7A1 (the rate-limiting enzyme for the classical pathway of BA synthesis) by activating SHP. SHP further binds to LRH1. In the intestine, FXR suppresses CYP7A1 expression by upregulating FGF15 (FGF19 in humans) expression. FGF15 further binds to the FGFR4/β-klotho complex and induces JNK1/2 and ERK1/2 signaling. BAs, bile acids; TCA, taurocholic acid; GCA, glycocholic acid; TCDCA, taurochenodeoxycholic acid; GCDCA, glycochenodeoxycholic acid; BSH, bile salt hydrolase; DCA, deoxycholic acid; UDCA, ursodeoxycholic acid; LCA, lithocholic acid; FXR, farnesoid X receptor; SHP, small heterodimer partner; LRH1, liver receptor homolog 1; FGF15, fibroblast growth factor 15; FGFR4, FGF receptor 4.

The gut microbiota also regulates BA metabolism through the nuclear farnesoid X receptor (FXR) (Jia et al., 2018; Sun et al., 2021) (Figure 1). FXR is a crucial BA receptor (the other is membrane G protein-coupled receptor 5, TGR5) and is closely related to CRC and HCC. Unconjugated BAs (e.g., chenodeoxycholic acid, CDCA; deoxycholic acid, DCA; lithocholic acid, LCA; and cholic acid, CA) are bacteria-produced high-affinity agonists of FXR. The rank order for the ability of BAs to activate FXR is CDCA > DCA > LCA > CA. FXR levels are closely correlated with CRC and HCC. Several FXR disruption studies have confirmed the role of FXR in the initiation of colon or hepatocellular carcinogenesis. In a CRC mouse model, whole-body FXR depletion resulted in increased expression of inflammation-related genes, lymphoid nodule numbers, and intestinal crypt heights, as well as fewer differentiated goblet cells. These morphological and genetic changes reveal an increased susceptibility for CRC in the mouse model (Modica et al., 2008). Similarly, *Fxr*-null mice spontaneously developed hepatocellular adenomas and carcinomas with increased circulating and hepatic BA levels, pro-inflammatory cytokines, and myelocytomatosis oncogene (Yang et al., 2007). In contrast, overexpression of FXR by adenovirus injection inhibited xenograft growth in nude mice. Colon cancer cells co-cultured with FXR agonists showed suppressed cell proliferation (Peng et al., 2012).

The mechanism of FXR-regulated intestinal carcinogenesis has not been clearly defined but may involve interactions with matrix metallopeptidase 7 (MMP7) (Peng et al., 2019) or innate immunity-related molecules (Vavassori et al., 2009). The mechanism of FXR in HCC initiation depends on the FGF15/19–FGFR4–β-klotho axis. Abnormal activation of this axis directly stimulates the epithelial–mesenchymal transition of HCC cells, resulting in invasion and metastasis in an HCC mouse model (He et al., 2015). In particular, increased hepatocellular FGF19 levels were positively correlated with cirrhosis in patients with HCC, whereas depletion of FGF15 inhibited hepatocellular proliferation *via* tumor suppressors such as *Ndrg2* (Deuschle et al., 2012) and *miR-122* (He et al., 2015).

#### Bacterial components

Inflammation also can be induced by bacterial components, especially lipopolysaccharide (LPS). LPS is a cell wall component of Gram-negative bacteria that induces inflammation through interactions with pattern recognition receptors (PRRs). PRRs are expressed on the surface of most innate immune response-related cells. Toll-like receptors 4 (TLR4) are PRRs that recognize LPS and activate NF-κB through myeloid differentiation factor 88 (MyD88)-dependent or MyD88 adaptor-like (MAL) pathways. A cross-region cohort study analyzing 526 metagenomic fecal samples from CRC patients and healthy controls identified several LPS-related signaling pathways (Dai et al., 2018). Seven dominant bacterial species may contribute to those pathways, including *Bacteroides fragilis*, *F. nucleatum*, *Porphyromonas asaccharolytica*, *Parvimonas micra*, *Prevotella intermedia*, *Alistipes finegoldii*, and *Thermanaerovibrio acidaminovorans*. Similarly, in a case–control study with 139 HCC patients and matched controls, levels of serum anti-LPS antibody and anti-flagellin Ig A and IgG were significantly higher in the HCC group (Fedirko et al., 2017). This tendency was positively correlated with an increased risk of HCC.

### Dysbiosis alters the immune response

The immune system helps identify and destroy nascent tumor cells and plays an important role in cancer defense. Dysfunction of the innate or adaptive immune systems induced by the bacteria-derived metabolites and bacterial components promotes carcinogenesis in the intestine and liver.

#### Evidence of gut microbiota in regulating immune response in CRC or HCC

GF mice without bacteria showed immature immune systems with poor gut-associated lymphoid tissue, lower levels of tissue-resident macrophages, smaller spleens, and decreased serum immunoglobulin levels. This incomplete immune system can be corrected by introducing intestinal bacteria, bacterial metabolites (e.g., SCFAs), or bacterial components (e.g., polysaccharide A, PSA) (Allen-Vercoe et al., 2011; Malik et al., 2018). Gut microbiota depletion by antibiotics alleviated the tumor burden in mice with CRC. However, this protective effect was not observed in mice with immune gene knockouts (Tao et al., 2007), indicating it depends on an intact immune system. In addition, exposure to gut microbiota stimulated the expression of CRC-associated chemokine genes (e.g., CCL5, CXCL9, CXCL10, CXCL1, and CCL20) (Cremonesi et al., 2018). Increased levels of CRC infiltrating-related chemokines helped inhibit CRC development by recruiting tumor-infiltrating lymphocytes (TILs). Therefore, patients with specific chemokine-expressing bacteria show improved survival rates. Similarly, treatment with *Akkermansia muciniphila* or Amuc_1100 (an outer membrane protein produced by *Akkermansia muciniphila*) improved colitis in mice with CRC by reducing levels of infiltrating macrophages and CD8^+^ cytotoxic T lymphocytes in the colon (Wang et al., 2020b).

#### Bacterial metabolite-based intestinal immune system

The gut microbiota influences the normal functions of innate and adaptive immune response through its metabolites, including SCFAs, aryl hydrocarbon receptor (AhR) ligands, and polyamines.

**FIGURE 2:**
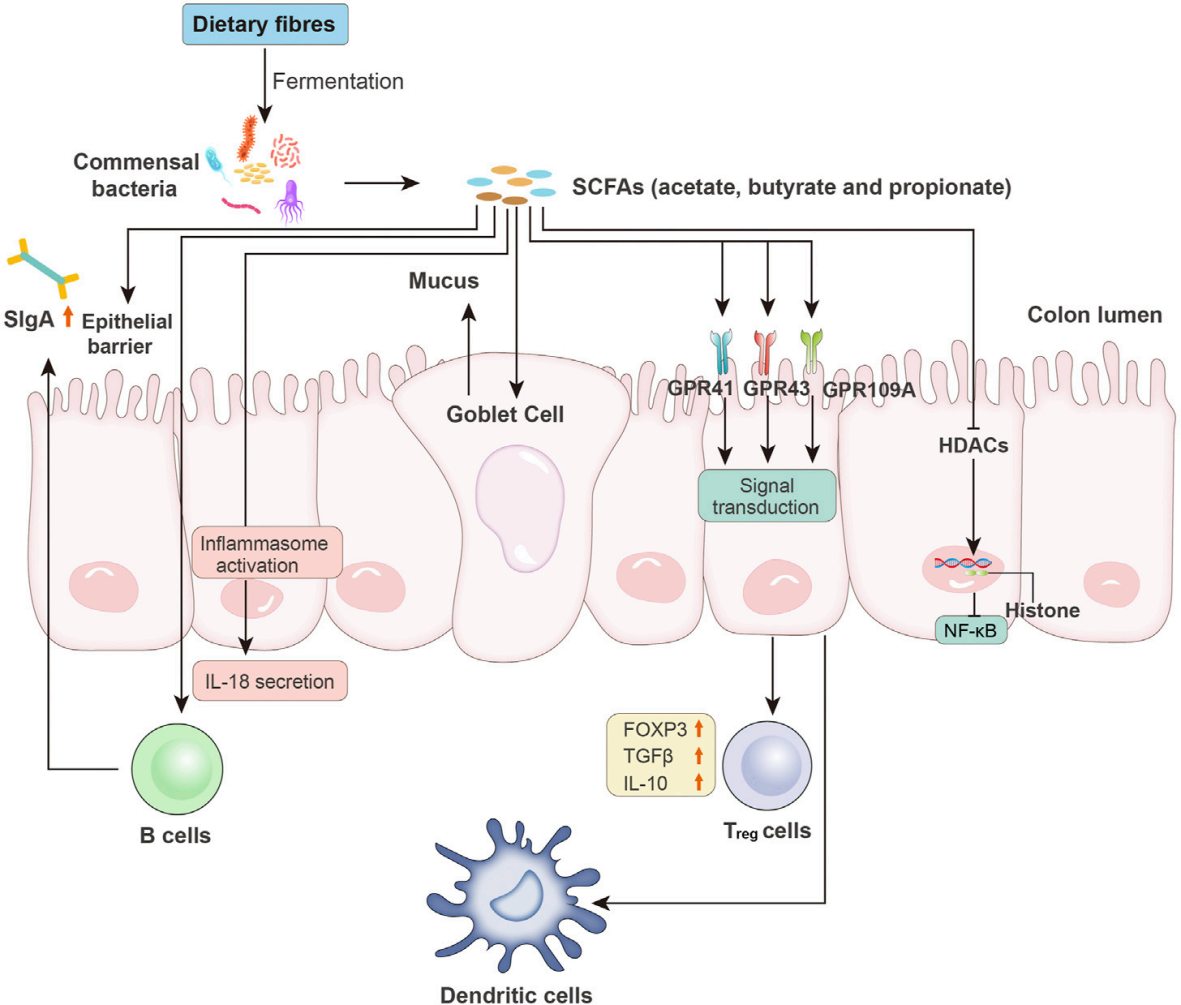
Microbiota-derived SCFAs and intestinal immunity. SCFAs, such as acetate, butyrate, and propionate, are produced by intestinal bacteria *via* the fermentation of undigested dietary fibers. SCFAs affect the host immune system through the following pathways: increased epithelial barrier function; increased B cell-based sIgA secretion; activation of inflammasomes and induced production of IL-18; enhanced goblet cell-based mucus production; binding to epithelial surface GPCRs (e.g., GPR41, GPR43, and GPR109A) to reduce expression of T-cell-activating molecules on antigen-presenting cells (e.g., dendritic cells) and increased levels of FOXP3 and anti-inflammatory cytokines (e.g., TGF-β and IL-10) in T_reg_ cells; and inhibition of HDAC activity, followed by NF-κB inhibition. sIgA, secretory IgA; GPCRs, G protein-coupled receptors; FOXP3, forkhead box P3; T_reg_ cells, regulatory T cells; HDACs, histone deacetylases.

SCFAs affect nearly every process of the intestinal immune response. Thus, they play important roles in maintaining the intestinal immune system (Figure 2). First, SCFAs serve as signaling molecules in the innate immune system by inhibiting HDACs (Rooks and Garrett, 2016). This inhibitory effect is NF-κB-dependent (Kendrick et al., 2010). HDAC suppression also facilitates the inhibitory activity of FOXP3^+^ regulatory T (T_reg_) cells (Tao et al., 2007). Signaling molecule GPCRs, including GPR43, GPR41, and GPR109A, are also involved in the SCFA-mediated immune response. Third, SCFAs help maintain intestinal barrier integrity. The commensal bacteria in the lumen (bacterial barrier), immunoglobin A (IgA) secreted by intestinal immune cells (immunological barrier), and the mucus layer and epithelial elements (physical barrier) help maintain intestinal integrity and reduce intestinal permeability. This protection wall efficiently prevents lumen colonization by pathogens and the translocation of bacteria or their products and components, such as LPS. Increased gut permeability or the so-called leaky gut is closely related to the onset of CRC and HCC. Exposure to LPS in the liver activates Kupffer cells *via* binding to TLR4, resulting in increased TNF-α, IL-6, and IL-8 levels. The increased production of proinflammatory cytokines and chemokines leads to HCC, as discussed previously and elsewhere (Yu et al., 2010; Dapito et al., 2012; Sanduzzi Zamparelli et al., 2017). Similarly, increased barrier function is helpful for CRC treatment (Bhutiani et al., 2018). Exposure to SCFAs increased the expression of mucin genes in epithelial goblet cells and strengthened the immunological barrier *via* mucosal immunity (Willemsen et al., 2003; Gaudier et al., 2004). SCFAs also modulate the permeability of tight junctions in intestinal epithelial cells, which is an important element in the physical barrier. A compact tight junction inhibits the translocation of enteropathogenic toxins. Colonization with SCFAs-producing bacteria relieved mice from infection by *E. coli* (Fukuda et al., 2011).

Aryl hydrocarbon receptor (AHR) is expressed by immune cells, epithelial cells, and some tumor cells. It is a member of the periodic circadian protein (PER)-AHR nuclear translocator (ARNT)-single-minded protein (SIM) superfamily of transcription factors. AHR is activated by both endogenous (e.g., kynurenine, a tryptophan derivative) and exogenous factors (e.g., diindolylmethane and indolocarbazole derived from the diet) (McIntosh et al., 2010). These factors are known as AHR ligands. The binding of AHR to microbial metabolites, such as the indole derivatives of tryptophan (Cervantes-Barragan et al., 2017; Carambia and Schuran, 2021), plays an essential role in the regulation of innate and adaptive immune responses. Deficiency of AHR or AHR ligands in mice led to increased *Bacteroides spp.*, decreased antimicrobial peptide (AMP) production, and reduction of intestinal intraepithelial lymphocytes (IELs) and turnover of intestinal epithelial cells (IECs) (Li et al., 2011). Injection of wild-type IELs to *Ahr* ^−/−^ mice with a maintained IEC barrier function and normalized bacterial composition. In wild-type mice, deficiency of AHR ligands resulted in increased severity of dextran sodium sulfate-induced colonic inflammation. The inflammation was improved when the mice were administered AHR ligand-containing diets. Together, these studies provided solid evidence for the AHR-mediated immune response. In general, indoleamine 2,3-dioxygenase 1**–**tryptophan and 2,3-dioxygenase 2**–**kynurenine**–**AHR (IDO1/TDO2**–**KYN**–**AHR) are important axes in AHR-involved immune regulation. IDO1 and TDO2 are two intracellular heme-containing metalloproteins that convert tryptophan into a series of biologically active molecules, such as KYN and several indole-containing substrates (e.g., neurotransmitter melatonin). The KYN pathway is the major route of tryptophan intake. KYN and its metabolic product kynurenic acid are endogenous AHR ligands (Gaitanis et al., 2008; Lamas et al., 2016). KYN-activated AHR is an important regulator of both innate and adaptive immune cells. Its activation can lead to the generation of immune-tolerant dendritic cells (DCs) and T_reg_ cells, resulting in a host-friendly tumor microenvironment (TME). This action is essential for the eradication of cancer cells (Cervantes-Barragan and Colonna, 2018; Cheong and Sun, 2018).

Polyamines such as spermidine, spermine, and putrescine are polycationic molecules produced by almost all living cells, including bacteria. The human intestinal lumen contains high levels of polyamines, which are derived from the diet and metabolism by the host and bacteria. Gut bacteria can use amino acid decarboxylase to produce polyamines that differ from the mammalian versions (using the arginase 1 and ornithine decarboxylase). Polyamines can affect the virulence of bacterial pathogens and also strengthen the integrity of the IEC barrier. Polyamines can induce the production of intercellular junction proteins (Rao et al., 2020), including zonula occludens 1 (ZO1), occludin, and E-cadherin, which are vital for maintaining intestinal permeability and enhancing epithelial barrier function. Moreover, polyamine metabolism plays an important role in regulating both innate and adaptive immune responses. Spermine can suppress the classic (M1) macrophage activation by inhibiting the expression of ornithine decarboxylase and the synthesis of pro-inflammatory cytokines (Kaczmarek et al., 1992; Zhang et al., 2000). The administration of *Bifidobacterium animalis* subsp. *lactis* LKM512 with an arginine-containing diet led to decreased levels of colonic TNF and IL-6 (Kibe et al., 2014). Pups supplied with polyamine showed elevated maturation of lamina propria CD4^+^ T cells and intraepithelial CD8^+^ T cells, accompanied by an earlier appearance of splenic B cells (Pérez-Cano et al., 2010). Higher polyamine concentrations are generally associated with carcinogenesis (Casero et al., 2018). Patients with cancer have increased polyamine levels in their blood and urine compared to healthy individuals (Kassi and Kaltsas, 2022). The dysregulation of polyamine metabolism by the host or gut microbiota may contribute to CRC (Yang et al., 2019). Furthermore, polyamines can suppress anti-tumor immune responses. Polyamine depletion *via* inhibition of ornithine decarboxylase activity attenuated tumor growth in a T-cell-dependent manner (Hayes et al., 2014), supporting the hypothesis that reducing intra-tumor polyamines may reverse immunosuppression in the TME.

#### Bacterial components-based immune response

The gut microbiota also modulates the immune response through bacterial components, including formyl peptides, PSA, and peptidoglycan. Formyl peptides are conserved N-formyl peptide motifs secreted by bacteria including *Staphylococcus aureus*. High concentrations of formyl peptides activate formyl peptide receptor 2 (FPR2) (Bloes et al., 2015) and induce neutrophil diapedesis at infection sites. PSA is a well-known polysaccharide produced by *ETBF*. It works with TLRs on DCs and is presented to T cells by CD11c+ DCs. The anti-inflammation effect of PSA depends on IL-10-producing CD4^+^ T cells and IL-10-producing CD25^+^ FOXP3+ T_reg_ cells (Round and Mazmanian, 2010). Peptidoglycan has a harmful effect on nucleotide-binding oligomerization domain-containing protein 1 (NOD1). NOD1 signaling is related to the innate immune system, which increases the number of ileal γδ T cells and stimulates the release of pro-inflammatory cytokine IL-17A (Hergott et al., 2016).

### Dysbiosis-induced DNA damage *via* genotoxins

Gut microbiota-produced genotoxins also work as second hits that accelerate CRC and HCC carcinogenesis. Cytolethal distending toxin (CDT) and colibactin are two genotoxins that induce DNA double-strand breaks. CDT and CDTB (its active subunit) are mainly produced by *Campylobacter jejuni*. CDT can lead to genetic instability by inducing replicative stress in several human cells (e.g., HeLa, U2OS, and RKO) and human colorectal organoids (Tremblay et al., 2021). This replicative stress leads to chromosomic aberrations by slowing DNA replication and expressing fragile sites. In addition, mice treated with a CDTB mutant *Campylobacter jejuni* (*C. jejuni*) showed less DNA damage and a lower risk of CRC (He et al., 2019)*.* Interestingly, this CDT-mediated carcinogenesis was autophagy-dependent (Seiwert et al., 2017), indicating that gut microbiota-produced genotoxins may interact with the cancer-related cellular stress response. Similarly, *E. coli*-produced colibactin induced CRC in both mice and humans. A recent study identified a DNA-damage signature in bacteria-infected human colorectal cells (Dziubańska-Kusibab et al., 2020), which suggested the etiological role of colibactin in CRC.

### Dysbiosis induces cellular stress response associated with genetic, epigenetic, and/or neoplastic changes

#### Dysbiosis induces genetic and epigenetic alterations

Human intestinal organoids exposed to *pks + E. coli* show distinct mutational signatures, including increased numbers of single base substitutions (SBSs) and induced insertion–deletion (ID) (e.g., single T deletions at T homopolymers) (Cremonesi et al., 2018). These characteristic mutations have also been observed in human cancer genomes, especially in patients with CRC. Studies have proposed the underlying mechanisms for dysbiosis-involved carcinogenesis, i.e., the effects on gene mutation. A study on mutant *p53* further discussed the role of gut microbiota on gene mutation (Kadosh et al., 2020). In general, somatic mutations in *p53* inactivate its tumor-suppressor function and confer oncogenic gain-of-function. Model mice with mutant *p53* showed contrasting effects in different intestinal segments. An oncogenic effect was observed in the distal gut (colon and ileum) through activated WNT signaling, whereas a tumor-suppressive effect was observed in the proximal gut and tumor organoids. The administration of an antibiotic cocktail reversed the oncogenic effect of mutant *p53* in the distal part, with shorter crypts, better-organized villi, and decreased WNT signaling. These data suggested that microbiota in the distal part are crucial for the oncogenic effect of mutant *p53*. Gut microbiota help switch mutant *p53* from tumor-suppressive to oncogenic. Gut microbiota also can change the function of long non-coding RNA (lncRNA). *F. nucleatum*, the predominant bacteria in patients with CRC, stimulates glucose metabolism in CRC cells by activating the transcription of lncRNA enolase 1-intronic transcript 1 (ENO1-IT1) (Hong et al., 2021). This increased transcription further regulates the activation of KAT7 histone acetyltransferase, consequently altering the CRC biological function.

The gut microbiota is also associated with epigenetic events such as DNA methylation (Sobhani et al., 2020). DNA methylation is a crucial process in carcinogenesis. Exposure to gut microbiota induces methylome and transcriptome changes in intestinal epithelial cells and is beneficial for mild host inflammation within the mucosa. Thus, dysbiotic microbiota drives aberrant epigenetic events, leading to CRC or HCC. DNMT1, DNMT3B, and EZH2 histone (H3K27) methyltransferases are upregulated in microsatellite instability tumors in CRC (Joensuu et al., 2015).

#### Dysbiosis modulates cancer-related cellular stress responses

Autophagy (Onorati et al., 2018; Li et al., 2020) and oxidative stress (Greenwood and Witney, 2021; Kuo et al., 2022) are cancer-related cellular stress responses. Autophagy refers to a series of mechanisms that transport superfluous or potentially dangerous cytoplasmic entities to the lysosome for degradation. The inhibition of autophagy sensitizes cells to regulated cell death. Evidence supporting the involvement of gut microbiota in autophagy is as follows: first, several bacteria-produced proteins are mediated by autophagy receptors such as SQSTM1/p62 and CALCOCO2/NDP52 (Sudhakar et al., 2019). Second, autophagy-mediated inhibition of type I interferon is gut microbiota-dependent (Martin et al., 2018). Third, the administration of gut microbiota-produced metabolites (butyrate) increases the expression of LC3 (a marker for autophagy) and the formation of autolysosomes in human colorectal cells (Luo et al., 2019). Recent studies showed that exposure to pathogen-associated molecular patterns (PAMPs) in dysbiosis activated hepatic NOD2, a general intracellular PRR, *via* bacterial muramyl dipeptide (MDP). The NOD2 activation was receptor-interacting protein 2 (RIP2)-dependent and upregulated NF-κB, JAK2/STAT3, and MAPK signaling in hepatocytes to promote a carcinogenesis-related pro-inflammatory response in the liver. Moreover, RIP2-dependent NOD2 activation induced a novel nuclear autophagy pathway. After transport to the nucleus, NOD2 binds to lamin A/C, a component of nuclear laminae, resulting in its protein degradation, ultimately leading to impaired DNA damage repair and increased genomic instability (Zhou et al., 2021). Oxidative stress is another gut microbiota-associated cellular stress response. Microbiota-produced metabolites such as butyrate can induce the production of reactive oxygen species (ROS) in HCC cells (Pant et al., 2017a; Pant et al., 2017b). The gut microbiota also produces endogenous ethanol, which is metabolized into acetaldehyde and acetate. Ethanol and its derivates induce ROS formation (Zhu et al., 2018; Sha’fie et al., 2020) by hepatic stellate cells (HSCs) and Kupffer cells, resulting in increased TLR4 expression (Hubbard et al., 2015). The resulting production of inflammatory cytokines leads to liver injury, including HCC.

## Gut microbiota-based therapy in CRC and HCC

As discussed earlier, dysbiosis is the first hit for the digestive system. Therefore, reshaping the composition of commensal bacteria is an important method for cancer prevention and anti-tumor therapy in the digestive system, especially in CRC and HCC.

### The role of gut microbiota in the treatment of CRC and HCC

Most data on gut microbiota-based therapy has been reported in CRC, whereas data in HCC are limited. The gut microbiota was associated with the recruitment of T follicular helper cells (T_FH_) in both mouse models and patients with CRC (Roberti et al., 2020). T_FH_ cells are subtypes of tumor-infiltrating lymphocyte (TIL), the presence of which indicates a better prognosis. Increased family Fusobacteriaceae and decreased family Erysipelotrichaceae have been reported in patients with CRC. Increased Fusobacteriaceae harms TIL recruitment, thereby promoting colonic tumorigenesis. In contrast, some bacteria show beneficial effects on cancer therapy. In CRC-related immunotherapy, patients with efficient responses showed increased *Bifidobacterium* (Sivan et al., 2015; Matson et al., 2018) and *Faecalibacterium* (Gopalakrishnan et al., 2018). In addition, a lower risk of relapse is associated with decreased *F. nucleatum* in patients receiving post-neoadjuvant chemoradiotherapy (nCRT) (Serna et al., 2020). Similarly, mice gavage with *Bifidobacterium* showed an efficient response to anti-CD47-based immunotherapy (Shi et al., 2020). Ileac residence of *Bacteroides fragilis* and Erysipelotrichaceae was associated with an immunosurveillance-based protective effect in a mouse model of CRC (Roberti et al., 2020).

### Gut microbiota-based therapeutic approaches in CRC and HCC

The first approach involves the supplementation of indigestible fermentable dietary fibers (i.e., prebiotics) or living microorganisms (i.e., probiotics) to increase beneficial intestinal bacteria. Some studies mixed prebiotics and probiotics to form synbiotics. Oligosaccharides such as fructooligosaccharides (FOS) and galactooligosaccharides (GOS) are the most common materials used as prebiotics. The commonly used probiotics include *Bifidobacteria*, *Lactobacilli*, *Streptococcus*, and VSL#3. VSL#3 is a mixture of probiotics, which can increase beneficial bacteria such as Lachnospiraceae, *Faecalibacterium*, and *Ruminococcus* (Jena et al., 2020).

Fecal microbiota transplantation (FMT) is the second approach. It directly reverses the overall bacterial composition and is a clinic-favored strategy for dysbiosis-dependent cancer therapy. The potential effect of FMT on CRC and HCC is still under investigation, although it is a useful treatment for recurrent *Clostridium difficile* infection (CDI) (Hvas et al., 2019; Khoruts et al., 2021).

Regarding bacteria-produced metabolites, the administration of postbiotics (e.g., SCFAs) has been accepted as a third approach for indirectly managing gut microbiota-involved carcinogenesis. A recent consensus statement defined postbiotics as “the preparation of inanimate microorganisms and/or their components that confers a health benefit on the host” (Salminen et al., 2021). Different materials and components can be used as postbiotics, including cell-free supernatants from bacterial culture medium, bacterial exopolysaccharides, enzymes or cell wall fragments, bacterial lysates, and microbiota-produced metabolites (Żółkiewicz et al., 2020).

Similarly, paraprobiotics (or ghost probiotics) have also been introduced to amend dysbiotic microflora. Paraprobiotics refers to viable microbial cells or crude cell extracts that confer a benefit to the receiver (either human or animal) when orally or topically administrated (Taverniti and Guglielmetti, 2011). Several methods can be utilized to prepare inactive probiotics, including heat, gamma or ultraviolet rays, chemicals (e.g., formalin), and sonication.

In addition, other microbiota-targeted manipulation, such as supplementation of TLR antagonists (e.g., polymyxin B), FXR agonists (e.g., obeticholic acid), or prokinetics (e.g., cisapride), also serve as accessary approaches for HCC. The use of antibiotics is controversial. Antibiotics aim to suppress the overall growth of bacteria in the intestine and have demonstrated protective effects on tumor cell proliferation and invasion (Bullman et al., 2017; Han et al., 2020). However, antibiotics can cause further dysbiosis and are associated with CRC onset (Zhang et al., 2019).

Two recently updated reviews compared the advantages and disadvantages of each approach (Kaźmierczak-Siedlecka et al., 2020; Pothuraju et al., 2021). Some unsolved must be addressed for better clinical application. First, the use of probiotics may cause bacterial translocation and the transfer of resistant genes through horizontal gene transfer, especially in patients with underlying medical conditions. Similarly, FMT may transfer unrecognized pathogens from the donor to the recipient. However, aside from the safety issue, the potential benefits of FMT as a therapeutic strategy are much higher than those of probiotics. FMT improves the overall intestinal microbial diversity, whereas probiotics have limited bacterial input. The third inconclusive issue is postbiotics, since isolating metabolites synthesized by bacteria remains challenging. However, recently developed technology and analysis techniques, such as air-flow-assisted desorption electrospray ionization mass spectrometry imaging analysis (AFAI-MSI) (Chen et al., 2021), may help solve these problems.

## Conclusion and perspectives

In closing, evidence from preclinical models and clinical data support the “two-hit hypothesis” for dysbiosis and its associated alterations in the development of digestive system cancer. Dysbiosis alone is not powerful enough to potentiate carcinogenesis until the development of dysbiosis-induced alterations, including inflammation, aberrant immune response, gut microbiota-produced genotoxins, and cellular stress response associated with genetic, epigenetic, and/or neoplastic changes. These four mechanisms serve as second hits to speed up carcinogenesis in gastrointestinal cancer, especially CRC and HCC. Finally, three application-related questions remain to be addressed:

• Inter-individual differences in gut microbiota may complicate the differentiation of cancer patients and healthy controls. Therefore, is it possible to screen gut microbiota from healthy individuals and develop a list of “beneficial bacteria”? Moreover, can we build continent- or race-based lists of beneficial bacteria to distinguish healthy individuals from potential patients?
• Since each type of digestive system cancer harbors a unique gut microbiota, can we screen them to build a cancer type-based bacteria list? This category may help identify the “gut microbiota-based susceptible factors” in digestive system cancer and may be a novel measurement for cancer prevention.
• Dietary soluble fibers are fermented by gut bacteria into SCFAs, which have diverse health-promoting effects. Increasing numbers of studies have reported the beneficial role of SCFAs in cancer prevention and anti-tumor therapy. If possible, nutrition-based strategies should be incorporated into standard care for cancer therapy, as dietary interventions are both cost-effective and patient-friendly compared to other pharmacological interventions. In the future, standard criteria or guidelines for nutrient selection are needed and may improve treatment outcomes and tolerance of cancer therapy.
